# Structure and properties of nitrocellulose: approaching 200 years of research

**DOI:** 10.1039/d3ra05457h

**Published:** 2023-11-02

**Authors:** Edmund Morris, Colin R. Pulham, Carole A. Morrison

**Affiliations:** a School of Chemistry, EaStCHEM Research School, University of Edinburgh David Brewster Road, The King's Buildings Edinburgh EH9 3FJ UK c.morrison@ed.ac.uk

## Abstract

This review brings together almost 200 years of fragmented research on the structure of nitrocellulose to give an overview that covers production to application in composite materials. As a mouldable plastic, energetic rocket propellant and biomolecular binding membrane, nitrocellulose still finds widespread practical application today despite the inception of synthetic plastics. The influence of different cellulose source materials affects the structure and properties of nitrocellulose in ways that are not fully understood, and so this review brings together relatively recent developments in the understanding of cellulose nanostructures to highlight where the gaps in understanding now reside. The influence of nitration conditions on the material properties of nitrocellulose is described, together with the proposed mechanisms and equilibria associated with these synthetic routes. The reported crystal structures of nitrocellulose are also reviewed, and the confirmed structural features are separated from those yet to be proven. We also consider how nitrocellulose interacts with other compounds, to help explain the distinct properties of its composite materials. This review points to further work that is required to obtain well founded structural models of nitrocellulose, while highlighting opportunities to control and direct its structure to improve its material properties.

## Abstract 2

This review brings together almost 200 years of research on the structure of nitrocellulose to give an overview covering its production from cellulose through to application in composite materials such as propellants or biomedical tests.

## Graphical abstract

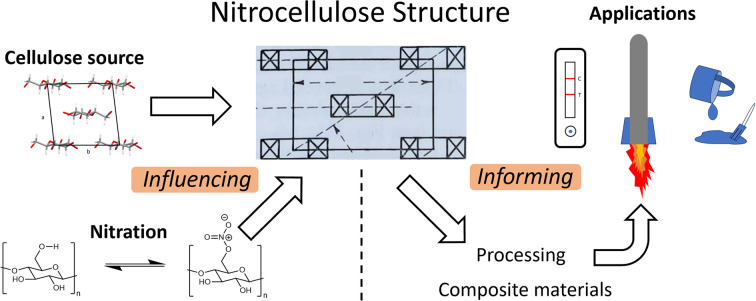

## 1 Introduction

As the first synthetic chemical derivative of cellulose, nitrocellulose (NC) has been studied for close to 200 years,^1^ with much interest in its properties as a mouldable plastic, lacquer, energetic binder and biomolecular binding membrane. The global NC market was valued at USD 0.86 billion in 2021 and is expected to reach USD 1.39 billion by 2030.^2^ Its solubility in organic solvents, mechanical strength and low affinity for water made NC an early plastic credited with leading the development of the modern plastics industry,^3^ with initial applications in camera film, billiard balls and adhesives. In addition, it is an energetic polymer, where the presence of both oxidising and reducing functional groups allow NC to burn without the need for atmospheric oxygen.^4^ While this property ultimately led to NC being replaced in some applications, the discovery of an energetic material with plastic properties made it ideal for rocket- and gun-propellant formulations. The combination of mechanical strength and ability to bind to other components make it the foremost energetic binder used in solid rocket propellants to this day.

**Fig. 1:**
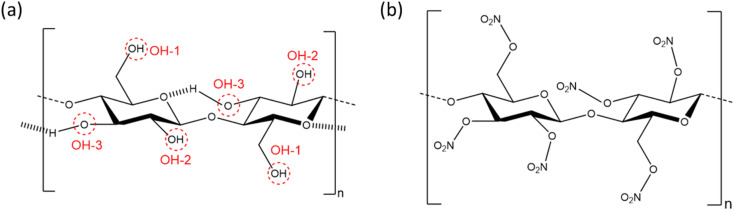
Repeating dimer units of (a) cellulose and (b) NC. The dimer illustrates the intra-chain interactions in cellulose, while the glucose monomer is the more accurate chemical repeating unit. OH-1 and OH-2 represent the two most accessible hydroxyl groups on cellulose for substitution, while OH-3 is the least accessible.

NC comprises glucose residues linked by the β(1–4) glycosidic bond, with the three hydroxyl group positions substituted by nitrate esters to varying degrees, as determined through the nitration process (see Fig. 1).^5^ The degree of substitution of one to three OH-groups is often expressed as percentage mass nitrogen or degree of nitration (DoN), and varies from 0% to a theoretical maximum of 14.14%.^6^ The three hydroxyl groups in cellulose are responsible for strong inter- and intra-chain hydrogen bonding interactions, leading to cellulose being a highly para-crystalline material with short-range molecular order.^7^ This is disrupted in NC, thus making DoN a key parameter in directing solubility, mechanical strength and energetic properties of the resulting material. NC with low-to-medium DoN finds application in lacquers, as it forms colloidal suspensions with common organic solvents to give viscous liquids that harden into strong, water-resistant coatings.^8^ NC with medium DoN is used in blotting membranes as a stationary phase for biomedical tests, such as lateral flow antibody tests, because the wettable microporous structure has a high affinity to interact and adsorb biological material, such as antibodies, which can then be immobilised in test lines.^9^ NC with high DoN finds applications in solid rocket propellants, where interactions with additional energetic components such as nitroglycerin create highly energetic composite materials, but which are chemically stable over extended periods of time.^4^

Although varied, the uses of NC all rely on a combination of its desirable mechanical properties and its ability to interact with other materials (*e.g.* solvents, proteins or oxidisers). However, despite nearly 200 years of research, many fundamental structural features of this important material remain only partially understood. Much of the early work is reported in hard-to-access journals, and as a consequence some details are routinely reported as facts when the original data does not warrant this status. While there are several reviews^8,10^ and chapters^11,12^ covering NC over the last 30 years, none are on the structure of NC. Hence, the purpose of this review is to assess critically the current understanding of the structure of NC, and how this is impacted by the identity of the cellulose source and the nitration process. We also draw parallels with the recent advances in understanding the nanostructure of cellulose and show the relevance to NC. Further discussion on the interactions of NC in composite materials will also be presented, along with new developments in controlling the properties of this important industrial material.

## 2 Preparation of NC

The widely varying properties of NC are determined by two main factors: (i) the initial cellulose source and (ii) the method of nitration.^6^ Both influence the structure of NC which in turn adds to its complexity, giving diversity which leads to the broad material applications that NC finds. While much work has been devoted to exploring the chemical conditions required to produce NC with consistent properties, this is far from a solved problem. The influence of the cellulose source is a significant source of variation in NC in ways which are poorly understood.^6^ Incomplete investigations into the preparation methods and initial sources of cellulose have created gaps in knowledge at a level which is unusual in modern commercialised materials with such significant applications, leaving the manufacture of NC disproportionately exposed to natural variation and supply-chain issues.

### 2.1 Sources and composition of cellulose

As the most prevalent organic polymer on earth, cellulose is produced by plants, algae, tunicates (invertebrate animals) and bacteria, and as a result the structure, morphology and material properties can vary greatly.^13^ This is significant for the preparation of NC as the structural properties of the cellulose source are known to directly influence those of the nitrated product. For example, it is known that highly crystalline samples of cellulose produce more crystalline NC,^14^ and highly anisotropic sources of cellulose, such as ramie fibres, produce NC with similarly aligned crystalline domains.^15^ Thus, in order to understand the structure of NC, understanding the structure of cellulose is a good place to start. Cellulose is, however, a much more intensely studied material with many structural features of cellulose being well established, although some only relatively recently.^16^ In contrast, NC received most attention around 80 years ago,^6^ and attempts to date to create structural models for NC based on cellulose have delivered fairly limited outcomes.^17^ As such, there is room for new developments in our understanding of cellulose to be applied to NC.

**Fig. 2:**
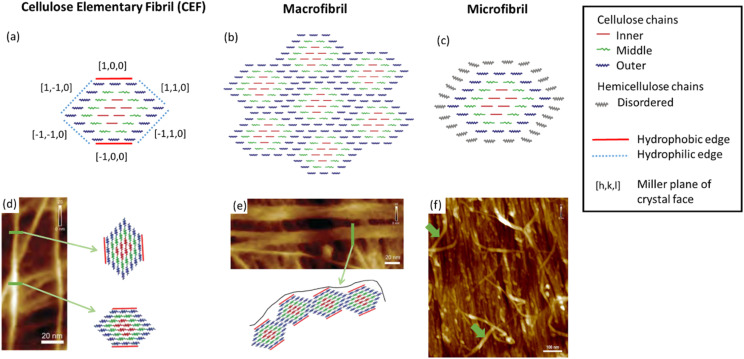
Illustrations of (a) a cellulose elementary fibril (CEF), (b) a macrofibril and (c) a microfibrils compared with their respective atomic force microscopy (AFM) images (d), (e) and (f). In (d) to (f) the green arrows highlight the structural feature illustrated. Illustrations adapted from ref. ^19^ with permission from American Chemical Society, copyright 2006 and AFM images reproduced from ref. ^22^ with permission from Springer Nature B.V., copyright 2014.

Cellulose is a semi-crystalline material with long thin crystalline domains, and its structure has been the subject of a recent review.^18^ These crystalline regions can be combined with amorphous cellulose and other macromolecules such as hemicellulose, lignin and pectin, which vary depending on whether the cellulose is algae- or plant-based. The structure of cellulose can be divided into three features, as illustrated in Fig. 2. Cellulose elementary fibrils (CEFs) form the most fundamental structural features in the crystalline domains, which for plants and many algae have a cross-sectional area of around 3 nm × 5 nm (the exact dimensions remain disputed) and lengths of up to several micrometres.^16,19^ With lengths almost a million times greater than their widths, disorder in CEFs occurs along this dimension, with experimental measurements estimating that crystallinity is disrupted every 150 nm or 300 glucan residues.^20^ Disorder along the fibril axis is important as it imparts greater flexibility to the cellulose fibres, as well as increasing the propensity towards hydrolysis. The CEFs then form one of two secondary structural features, which are known as micro- and macro-fibrils.^19^ The former are singular CEFs aggregated with hemicellulose, a shorter branched polysaccharide that is otherwise chemically identical to cellulose, and other polymeric compounds to a lesser extent. Macrofibrils are CEFs conglomerated into bundles, which arise due to hydrogen-bonding interactions between the CEF hydrophobic and hydrophilic edges (see Fig. 2).^21^ These are wider, sometimes ribbon-like, and more crystalline, than microfibrils.^22^

Plant cells produce cellulose which is deposited on the outside of the cell to construct the cell wall.^16^ Cellulose biosynthesis varies by organism, but for plant cellulose this process is performed by several associated proteins, known as cellulose synthase complexes, which are believed to produce CEFs of 18–36 polymer chains.^23^ The deposited cellulose changes in size, shape and crystallinity over the course of cell development.^24^ Features such as the size and shape of both CEFs and macrofibrils are known to vary during cell development, the most notable difference being that between the primary and secondary walls. Although the mechanism of this is as yet unexplained, recent progress in genetic sequencing of plants,^25^ combined with protein structure determination,^26^ shows promise in understanding the mechanistic function of the cellulose synthase complexes which drive CEF formation. Variation in the purity and structure of the plant cellulose could clearly influence the NC product, highlighting the poorly characterised relationship between plant development and NC produced from such plant sources.

Various cellulose sources have been used in the production of NC, with cotton fibre and wood pulp as the main commercial sources.^6^ As lignin and other impurities are thought to lead to poor stability of NC for long term storage, the naturally high purity of cotton cellulose makes it a practical NC precursor. Wood pulp requires additional purification and processing steps.^27^ As the structural features of cellulose vary in line with cell development, in practice cotton-fibre strengths will vary with harvest times, growth rates and temperature differences during growth.^28^ This inherent variability in a natural product is significant for the production of NC, especially for applications where reproducible and reliable mechanical properties are critical.

**Fig. 3:**
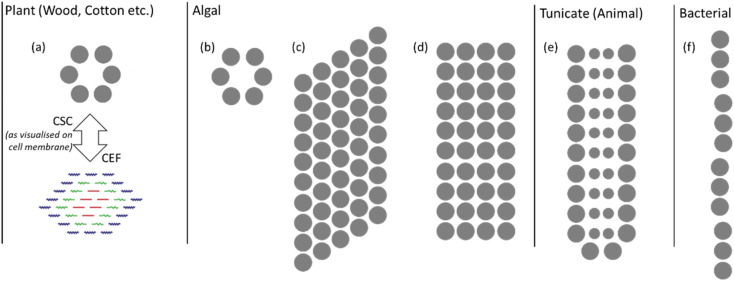
Illustration of CSCs for plant and BAT sources; the grey dots indicate where cellulose chains are extruded from the cell membrane. The plant CSC in (a) is compared with the CEF to show the relationship between their size and shape. Algal cellulose CSCs of (b) green algae (*Micrasterias*), (c) yellow-green algae (*Vaucheria*) and (d) red algae (*Erythrocladia*). The CSCs for (e) tunicate (*Metandroxarpa uedai*) cellulose and (f) bacterial (*Acetobacter*) cellulose. Illustration adapted from ref. ^29^ with permission from Taylor and Francis, copyright 1996.

The variation in and between plant sources is mostly confined to changes in morphology and proportions of cellulose to other macromolecular components. However, bacterial, algal and tunicate (BAT) cellulose sources vary fundamentally, with significant differences in the cellulose synthase complex (CSC), which results in different nanostructures. These are illustrated in Fig. 3 with the grey dots indicating the regions where cellulose chains are extruded. The arrangement of cellulose-producing proteins on the cell wall determines the arrangement of cellulose chains into the CEFs.^18^ While plant sources tend to have hexagonal CEFs, BAT sources can have much more oblong cross-sections as indicated by their CSCs. Notably there is much greater variation in CEFs for non-plant cellulose sources, with algae for example showing at least three different nanostructures.^29^ Furthermore, the purity of these sources can be much greater with fewer other macromolecular compounds produced on and around the cellulose fibrils. BAT sources therefore have different cellulose nanostructures and such variation in the building blocks of the fibres has been found to strongly influence macroscale properties such as Young's modulus.^30^ Understanding the nanostructure of non-plant cellulose sources remains an active area of research and much of this new knowledge is yet to be transferred to the study of NC.

With high variability in cellulose production arising from plants, as well as the promising properties of alternative cellulose sources, interest exists in switching to a BAT source. To date, work has almost entirely focused on bacterial cellulose,^31^ with strains such as *Acetobacter xylinum* shown to produce cellulose under controlled conditions (incubation time, temperature, pH and substrate, *etc.*), limiting the possibility for structural variation.^32^ Furthermore, cellulose produced by bacteria is also free from lignin and hemicellulose that are present in plant-based sources, making the resulting cellulose typically higher in crystallinity.^32^ Finding use as a foodstuff^33^ there are established processes for the purification and scale-up of bacterial cellulose,^34^ potentially making it an ideal source of cellulose for the manufacture of NC.

**Fig. 4:**
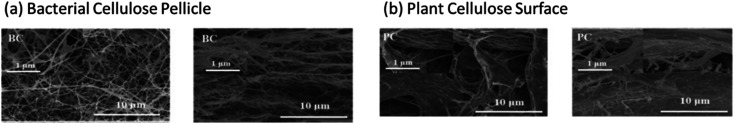
Scanning electron microscopy (SEM) images of (a) a bacterial cellulose pellicle and (b) plant cell wall surface of cotton cellulose. Images reproduced form ref. ^36^ with permission from MDPI, copyright 2019.

Compared with plant sources, which generally have some degree of uniaxial orientation of the polymer chains along the fibre or stem, bacterial cellulose is extruded by cells along multiple axes to form a mesh known as a pellicle (see Fig. 4). This could allow the production of NC with less anisotropy and therefore greater homogeneity. Electron microscopy images, however, do show the mesh-like morphology being preserved in the pellicle as can be seen in comparison with plant secondary cell wall cellulose in Fig. 4.^35^ For cellulose this morphology, combined with the high crystallinity, is known to impart a Young's modulus many times higher than that of plant sources. While the chemical and energetic performance of NC from bacterial cellulose has been characterised,^31,36^ the more significant mechanical properties such as glass transition temperature and tensile strength remain under-reported. As such, the potential for bacterial cellulose as a source for the preparation of commercial NC is yet to be fully explored but shows the potential to outperform current plant-based NC.

Algal and tunicate cellulose have been structurally characterised because of recent interest in cellulosic nanomaterials.^18^ Algal cellulose sources can exhibit wide variation in CEF, depending on the species of algae, which results in a variety of cellulose nanostructures. This could allow the optimum nanostructure to be chosen for each NC application, but there are currently no reports of NC synthesis from algal cellulose and so the properties of algal NC are unknown. With the great abundance of algae and scalability of their cellulose purification,^37^ algal cellulose shows potential as a sustainable cellulose source. Tunicate cellulose, the only animal source of cellulose, is thought to be a relatively plentiful resource, but isolation of cellulose gives a poor yield (31% of dry weight).^38^ While tunicate cellulose may be confined to specialist uses, it could aid the structure determination of crystalline NC; tunicate cellulose was used to determine the structure of the cellulose I_β_ polymorph.^39^ Irrespective of whether or not BAT cellulose sources ultimately find applications in NC production, understanding the impact of structural variation here is a valuable tool for understanding the potential for variation in the nanostructure of NC.

With a wide variety of cellulose sources displaying a large number of structural features, the optimisation and tuning of NC through tighter control of cellulose growth is very much a viable option worthy of further exploration. This, however, relies on not only characterising the structure of cellulose across numerous cellulose sources, a task which has seen significant progress in recent years,^40^ but also in establishing a firmer relationship between the structural features of cellulose and how they are translated into those of NC.

### 2.2 Nitration methods

**Fig. 5:**
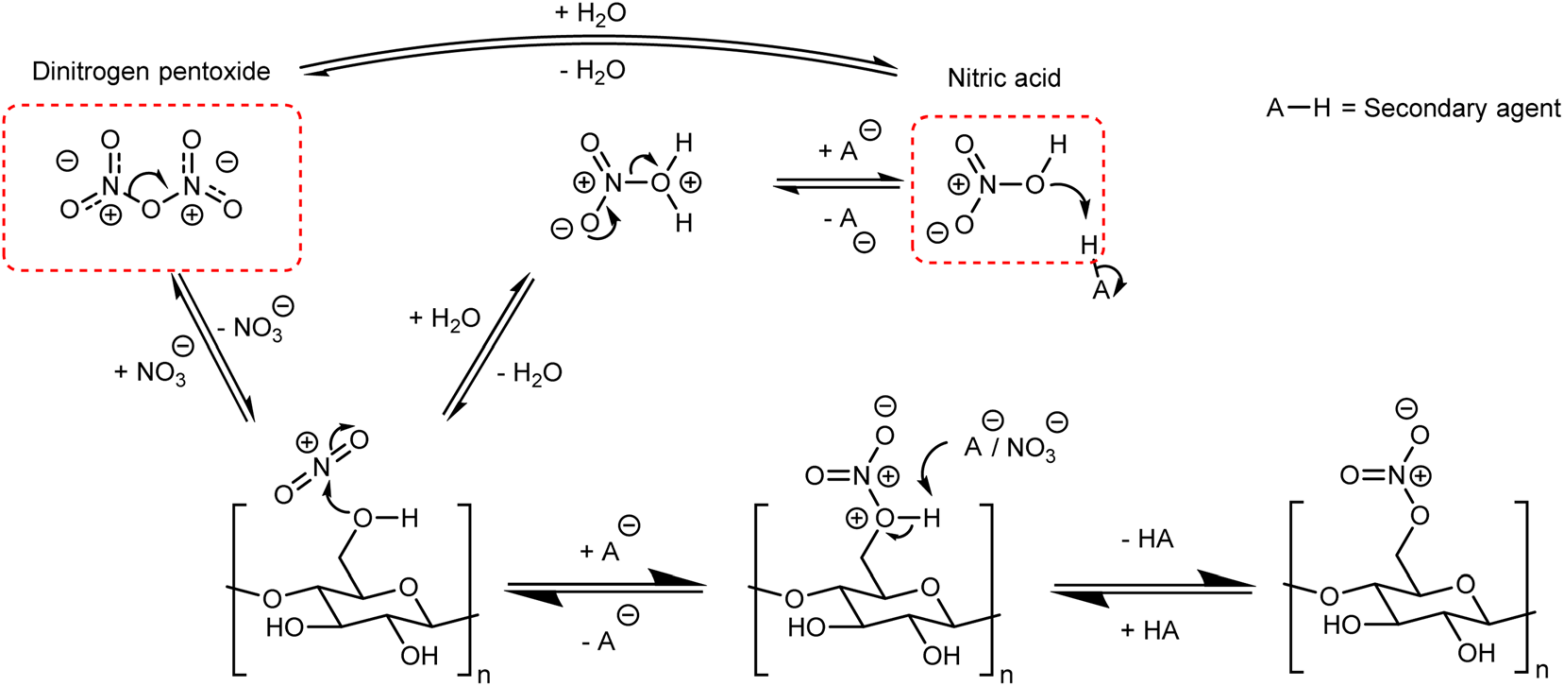
General mechanism for the nitration of cellulose.

Although there are many methods for the nitration of cellulose, the principle behind them is straightforward and universal. It can be simplified to the reaction of nitric acid with some or all of the –OH groups on the cellulose backbone. In order to form a nitrate ester on cellulose, the nitronium ion (NO_2_^+^) must be formed and attack one of the three –OH positions shown in Fig. 1;^41,42^ this general mechanism is outlined in Fig. 5. Almost all nitration methods use a combination of nitric acid and a secondary agent, which is typically a mineral acid such as sulfuric or phosphoric acid, that acts as a dehydrating agent to increase the concentration of NO_2_^+^.^5^ While there are reports of using dinitrogen pentoxide to directly nitrate cellulose,^6^ in the condensed or liquid form N_2_O_5_ ionises to NO_2_^+^ and NO_3_^−^ and therefore follows the same mechanism.^43^

The nitration of cellulose, while simple in principle, is complicated by interacting factors that change the equilibria highlighted in Fig. 5, and therefore influence the rate of reaction. One of those factors is the structure (and therefore the source) of cellulose. The macromolecular nature of cellulose requires shift and expansion of the inter-chain spacing during nitration. Recent work has pointed to this molecular motion being the rate limiting step for the nitration of cellulose and suggests strong links with the nanostructure of cellulose.^44^ In cellulose the CEFs are slightly distorted due to an imbalance in the strength of intra-molecular and inter-molecular hydrogen-bonding, giving a slight right-handed twist.^45^ It was proposed from X-ray diffraction and microscopy experiments that this untwisting of the microfibrils was the rate limit step for the nitration of cellulose.^46^ As the degree to which the fibrils are twisted relies on the precise shape of the CEFs this establishes a fundamental link between cellulose nanostructure and the rate of nitration, though the precise relationship between morphology, nanostructure and the rate of reaction has yet to be quantified.

The role of the secondary nitrating agent is largely understood to impart three important control features. These are (i) swelling of the cellulose fibres, to promote penetration of NO_2_^+^ deep within the cellulose structure,^47^ (ii) competing side reactions, leading to impurities and lower DoN, and (iii) hydrolysis, which reduces the polymer chain length.^48^ These key properties will be used to understand the most notable preparative methods for NC described below.

Mixtures of nitric and sulfuric acid are by far the most common nitration method, and are used in commercial processes.^6^ As the general scheme in Fig. 5 indicates, the proportion of nitric to sulfuric acid is a significant parameter to consider.^48^ In general, increasing the proportion of sulfuric acid increases the DoN of the product, but at proportions higher than three stoichiometric equivalents of sulfuric acid to nitric acid, the resulting DoN begins to decrease. Further evidence that the concentrations and proportions of acids are key to the formation of nitrate esters can be found in early equilibrium experiments^5^ and measuring the quantities of dinitrogen pentoxide formed while achieving different levels of DoN.^49,50^

As cellulose is a fibrous material, only surface nitration of cellulose would be possible unless the fibres are swollen by the reaction mixture.^6^ Notably, concentrated sulfuric acid can act as a solvent for cellulose,^51^ forming strong interactions with the –OH groups and enabling the hydrogen-bonding interactions in cellulose to be broken. Through a recent crystallographic study of sulfuric acid/cellulose solvates, these interactions have been found to be similar to the trihydrate structure of sulfuric acid, with one hydroxyl group taking the place of a water molecule.^52^ These strong interactions can partially explain how sulfuric acid promotes faster nitration of cellulose by swelling the cellulose chains to enable more homogeneous nitration. In contrast, NC appears to interact much less strongly with sulfuric acid, with minimal swelling, hydrolysis and dissolution reported.^48^ While the nature of these interactions have not been well studied or explained in the literature, it is nonetheless clear that this accounts for NC's greater stability on exposure to strong acids, and the role of sulfuric acid in promoting homogeneous nitration.

The ability of sulfuric acid to hydrolyse the β(1–4) glycosidic bond^53^ and the relative ease with which cellulose forms sulfate esters,^54^ are often considered drawbacks for nitration in the presence of sulfuric acid. While it is often reported that they cause issues with the control of molecular weight and stability due to impurities in the resulting NC, these issues do not seem particularly inhibiting; nitration with sulfuric acid is, after all, the main commercial route to NC. The shielding effect of the nitrate groups limits the formation of sulfate esters and hydrolysis of NC.^55^ While there have been attempts to quantify sulfate ester formation in NC production through isotopic labelling,^56^ there have been no systematic studies on the influence of sulfuric acid catalysed hydrolysis on NC chain length. However, in order to enable developments in the production of NC where greater control of chain length (and hence mechanical properties) could be obtained, a systematic study on the degree of hydrolysis during nitration in the presence of sulfuric acid is required.

Nitration without sulfuric acid is primarily used to generate NC for research-level work, as it allows access to higher DoN levels that approach full substitution.^57^ The secondary agent is most commonly acetic acid/acetic anhydride or phosphorus pentoxide/phosphoric acid mixtures, which all provide strong dehydrating conditions when used at high concentration. Under these conditions the formation of NO_2_^+^ proceeds *via* dinitrogen pentoxide.^11,57,58^ As with sulfuric acid, higher stoichiometric proportions of these secondary agents leads to higher DoN, showing that control of DoN is maintained through the stoichiometric proportions of nitric acid to secondary agent.

The strongly oxidising and acidic nitration conditions that are employed yield a nitration product that must be stabilised before use. The cause of this instability is residual acid which has penetrated the cellulose structure and can subsequently catalyse nitrate ester bond cleavage;^3^ this bond has a relatively low activation energy (*ca.* 100–150 kJ mol^−1^, depending on the substitution position).^59^ Through stoichiometric and X-ray diffraction experiments, nitric acid was shown to intercalate between layers of NC chains in the crystalline regions, to form a trihydrate-like structure with water and remaining hydroxyl functional groups on the NC.^60^ While the exact structure of this material is likely to be disordered, making it hard to characterise, this is good evidence that nitric acid is present in the crystalline regions of NC. This both explains why NC is difficult to stabilise post nitration and potentially provides a mechanism for the heterogeneous nitration of cellulose through the formation of nitric acid hydrates bound to the hydroxyl groups on cellulose.

In industrial processes, NC stabilisation is typically achieved using a high-temperature water treatment.^6^ While this process is effective at preventing decomposition for several years, NC will still decompose over time, presumably through cleavage of nitrate ester bonds. Breaking these bonds generates NO_2_ which further accelerates the process, making the decomposition of NC autocatalytic.^61^ This is especially apparent on exposure to water, which increases the mobility of NO_2_ within the NC structure.^47^ Exposure to UV light is also thought to promote nitrate ester bond cleavage, as shown by studies on simple alkyl nitrates.^62^

To extend the safe lifespan of NC stabilisers based on aromatic amines (*e.g.* diphenylamine) or urea derivatives (*e.g.* 3-methyl-1,1-diphenylurea) are typically added.^63^ These are believed to function by preferentially binding the NO_2_ released from the NC, typically by undergoing a nitration reaction. While the decomposition pathway of NC is relatively well understood, variability in cellulose sources, and therefore the type and level of impurities present, has been shown to add additional levels of complexity.^27^

### 2.3 X-ray diffraction for the structure determination of NC

**Fig. 6:**
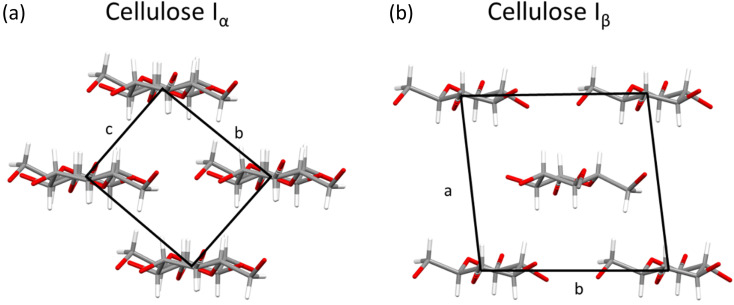
Crystal structures of (a) cellulose I_α_ viewed along *a* axis and (b) cellulose I_β_ viewed along *c* axis, with unit cells marked by black box. Plotted in mercury with structures from ref. ^64^ and ^71^ respectively.

While cellulose is a morphologically diverse material on the microscopic level, order is present at the nanoscale, where two naturally occurring polymorphs, known as I_α_ and I_β_, have been reported that differ only by a slight variation in chain conformation and hydrogen-bonding interactions (see Fig. 6).^39,64^ Most sources of cellulose will contain both polymorphs.^18^

**Fig. 7:**
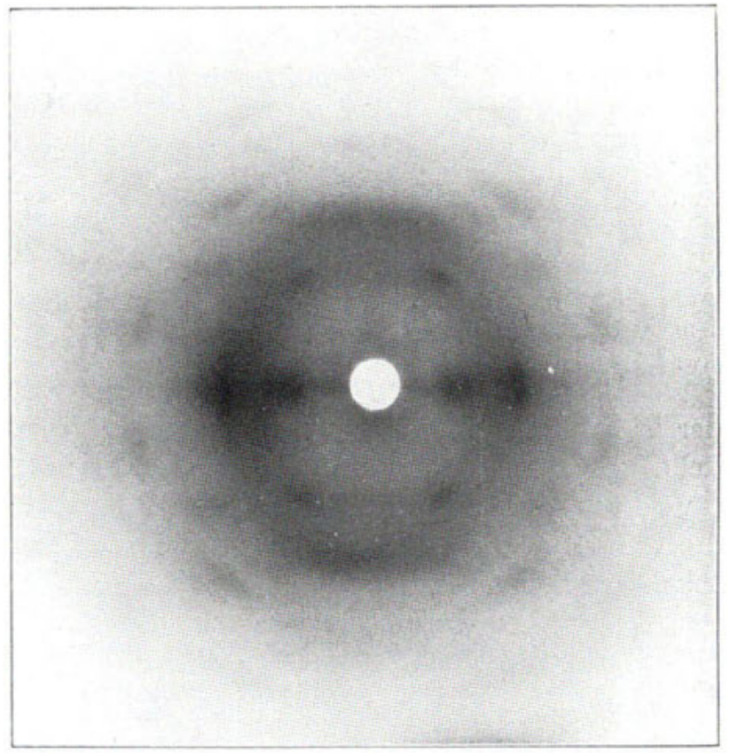
The first published fibre diffraction pattern of NC with DoN of 12.6%, derived from nitration of ramie plant fibres. Reproduced with permission from ref. ^68^ with permission from John Wiley and Sons, copyright 1927.

It has been proposed in multiple studies that partially nitrated NC is made up of a random arrangement of glucose monomers at varying degrees of substitution, which leads to diffuse diffraction patterns with a significant amorphous background.^65^ The earliest X-ray diffraction studies on NC, reported as far back as 1924,^66^ were conducted on samples prepared from ramie plant fibres, as these show uniaxial orientation of the crystalline domains and high levels of crystallinity.^67^ While cellulose has intra and inter-chain hydrogen bonding, these interactions are absent in NC chains (see Fig. 1). The weaker inter-chain interactions lead to broader diffraction patterns, as shown in Fig. 7 which dates back to work published in 1927.^68^

**Fig. 8:**
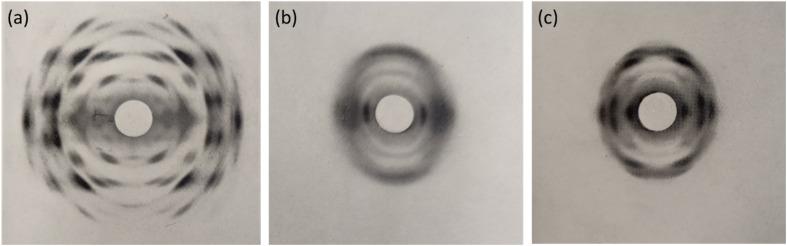
X-ray diffraction patterns of (a) a ramie cellulose fibre, compared with (b) with di-substitution NC and (c) tri-substitution NC. Diffraction patterns reproduced from ref. ^15^, copyright 1955.

Despite the poor quality data, these early diffraction patterns were qualitatively analysed to determine that the crystal structure of NC was structurally distinct from that of cellulose.^17^ Furthermore, these diffraction studies found that only fully nitrated NC shows a distinct crystalline structure, whereas lower degrees of nitration for which predominantly only functional groups OH-1 and OH-2 are nitrated (see Fig. 1), give more diffuse diffraction patterns.^68^ The existence of a distinct crystal structure relating to fully nitrated NC was also shown in the work by Miles, who reported a series of diffraction patterns relating to di- and tri-substitution.^15^ In comparison to the diffraction pattern of cellulose in Fig. 8(a), the di-substituted sample appears diffuse, while the fully nitrated samples gives rise to a pattern that regains crystallinity whilst appearing qualitatively distinct from the pattern from cellulose.

Over the intervening years there have been multiple attempts to propose a crystal structure for NC, with the results summarised in Table 1. Herzog compared the diffraction patterns of NC with cellulose to assign the Miller planes, and therefore proposed the first unit cell for NC.^66^ However, it was subsequently ascertained that the sample used in this study was only partially nitrated; in a follow-up publication Herzog and Náray-Szabó proposed a new unit cell for a sample confirmed to have a high DoN. This was the first reliable report of crystallographic unit-cell parameters for NC.^68^

**Table 1:** Proposed crystallographic unit cells for NC

Symmetry	Unit cell lengths/Å	Unit cell angles	Authors	Date	Ref.
Orthorhombic	*a* = 10.10	*α* = *β* = *γ* = 90°	R. O. Herzog	1926	^66^
	*b* = 8.56				
	*c* = 9.77 (fibre axis)				
Orthorhombic	*a* = 14.75	*α* = *β* = *γ* = 90°	R. O. Herzog and S. v. Náray-Szabó	1927	^68^
	*b* = 7.88				
	*c* = 10.30 (fibre axis)				
Orthorhombic^a^	*a* = 13.9	*α* = *β* = *γ* = 90°	M. Mathieu	1935	^69^
	*b* = 25.6 (fibre axis)				
	*c* = 9.0				
Orthorhombic	*a* = 12.4	*α* = *β* = *γ* = 90°	F. D. Miles	1955	^15^
	*b* = 25.4 (fibre axis)				
	*c* = 9.0				
Monoclinic	*a* = 12.3, or 14.6	*α* = *γ* = 90°, *β* = 63°	D. Meader, E. D. T. Atkins and F. Happey	1978	^70^
	*b* = 9.0				
	*c* = 25.4 (fibre axis)				

a While Mathieu described the unit cell as being monoclinic the *β* angle given was 90° therefore it could more accurately be described as orthorhombic or be monoclinic approximating to 90°.

Following on from the work by Herzog and Náray-Szabó, three subsequent studies report small changes in the unit-cell lengths. The *c*-axis, while initially disputed, was finally assigned to the fibre axis, and the length of *ca.* 25 Å was approximated to five monomer units (each *ca.* 5.2 Å).^47,69,70^ This, however, came from an assumption that NC would follow the same chain conformation of cellulose, which at that time was thought to have a 5-fold helical axis along the *c*-direction, stabilised by internal hydrogen-bond interactions.^70^ However, modern X-ray and neutron diffraction experiments have now shown this not to be the case.^39,64,71^ Thus, any assumptions based on the historic understanding of the conformation of the cellulose chain is unlikely to hold true for NC; moreover, NC cannot participate in the classical hydrogen bonding motifs originally postulated to stabilise the 5-fold helical structure. Cellulose I_α_/I_β_ and cellulose triacetate both show a two-fold screw axis (as represented in Fig. 1(a)), indicating the two-monomer conformation is a stable arrangement for the β(1–4) glycosidic bond and a favourable conformation for bulky substitutions on cellulose.^72^ Furthermore, the *c*-axis originally determined by Herzog *et al.* approximates to a 2-monomer NC repeat unit repeat along the chain.^68^ Thus at present there is a lack of consensus around the unit-cell parameters for NC and, while there is some indication as to the chain conformation, this also remains disputed.

**Fig. 9:**
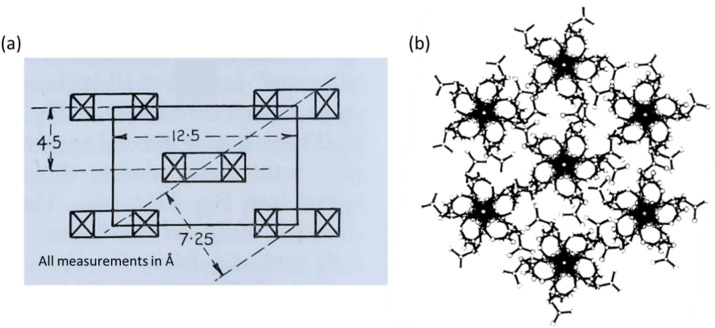
Crystallographic models of NC proposed by (a) Miles in 1955 (orthorhombic) and (b) Happey *et al.* in 1978 (monoclinic, showing the now disputed five-fold axis). Illustrations reproduced from (a) ref. ^47^, copyright Imperial Chemicals Industry 1955 and (b) ref. ^70^ with permission from Elsevier, copyright 1978.

The two proposed crystal structures for NC are shown in Fig. 9. Fig. 9(a) is based purely on diffraction data and does not show the chain conformation, whereas Fig. 9(b) attempts to show a fully atomistic model for NC, in which the lack of definition in the diffraction pattern was supplemented with coordinates obtained from computational modelling tools. The main difference between the unit cells from Miles and Happey *et al.* is a change in crystal symmetry from orthorhombic to monoclinic. Despite the drawbacks of each of the models, they do share some common elements, indicating that are some reliable features are known. Both are staggered, layered structures with inter-chain distances of around 7.5 Å, compared with 4.5 Å in cellulose. The structure also remains somewhat similar to that of cellulose, although with larger inter-chain spacings. This is consistent with bulkier nitrate ester groups and their corresponding weaker intermolecular interactions, while the remaining uniaxial orientation of the polymer chains is consistent with the structural influence of the cellulose source.

While some broad features of understanding can be gleaned from these early studies, the current crystallographic models lack detail, leaving the chain conformation unconfirmed as either a 5 or 2-fold helical axis. Such detail is important to understand intermolecular interactions and to provide a basis to explore structure–property relationships. More detailed crystal structures for NC would also provide a stronger basis for molecular dynamics (MD) simulations on NC. Here, recent work by Skylaris *et al.* has attempted to establish the structure and crystallinity of NC as a function of DoN.^73^ While the MD simulations fit at low DoN, where the underlying structure can be compared closely with that of cellulose, the agreement with experimental observations at high DoN were less reliable; this discrepancy could well be pointing to further limitations with the experimental crystal structure of NC.

### 2.4 Powder X-ray diffraction for the structural characterisation of NC

Atomistic structural models of NC have been held back by the difficulties associated with obtaining crystalline samples of NC of high enough quality for diffraction experiments. As an alternative technique, powder X-ray diffraction (PXRD) can give important structural information on individual NC samples, allowing for some level of characterisation and comparison.

**Fig. 10:**
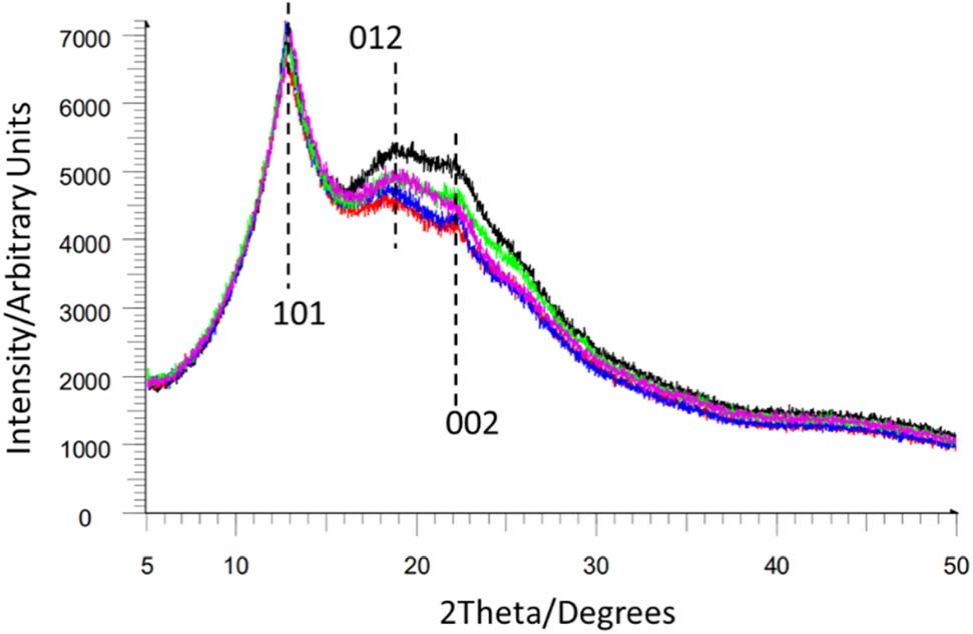
Multiple overlapping NC powder diffraction patterns from ref. ^74^ reproduced with permission from Fraunhofer ICT, copyright 2014, with Miller index planes marked from indexing of NC by Miles *et al.* from ref. ^47^.

While most NC samples give broad overlapping diffraction peaks (see Fig. 10) these still allow for the determination of crystallinity and estimation of the crystalline domain size *via* the Scherrer equation.^74^ This suggested that crystalline domains were between 58–90 Å in length in samples of NC mixed into composite materials such as gun propellants.^75^ Notably this crystalline domain size varied depending on the sample. Furthermore, the peak positions in the PXRD patterns of NC can be directly related to interlayer spacing (*ca.* 7.1 Å for Fig. 10, based on the [101] Bragg peak position, and cross-referencing to Miles' indexing of NC in Fig. 9). This in turn also permits calculation of the unit-cell density (approximately 1.9 g cm^−3^ for the samples in Fig. 10), giving a quantitative value for comparison of NC samples. PXRD can therefore be used to study the microstructure of NC and allows different NC samples to be quantitatively compared. For instance, Herrmann *et al.* used PXRD patterns to establish an inverse relationship between the viscosity and the degree of crystallinity for NC samples.^76^

## 3 Chemical interactions in NC

The chemical interactions exhibited by NC are key to its use and performance in a broad range of materials applications. In this section, we explore and summarise the body of work reported on NC's interactions with different components, including solvents, and show how this affects its structure and imparts its desired material properties.

### 3.1 Solvent interactions in NC

**Fig. 11:**
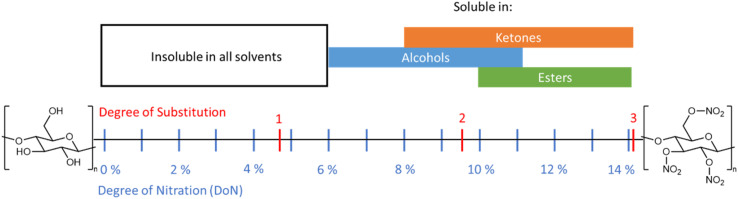
Schematic summarising the solubility of NC across a variety of functional groups in relation to DoN. Solubility data from ref. ^77^.

The solubility of NC in different media is a critical factor for industrial processing, and this in turn is directly affected by the DoN (see Fig. 11).^6^ NC across all DoN levels is insoluble in water, but NC with a mid-level DoN is miscible in lower polarity solvents, such as alcohols.^77^ At higher DoN this solubility drops further in favour of ester- and ketone-based solvents.^8,78^ Molecular chain length and crystallinity, and therefore the nature of the cellulose source material, can also influence the solubility of NC.^6^ This inherent variability often leads to issues in reproducing NC solubility, particularly with regard to binary solvent mixtures.^79^ Inhomogeneous NC, with a non-uniform nitrate ester substitution pattern, can also further contribute to these frequent disparities. Despite these challenges, the general trends in solubility of NC are well established; compared with the strong hydrogen bonding interactions presented in cellulose, the weaker interactions in NC generate a material that is more readily soluble in a variety of organic solvents.

The variable solubility of NC with respect to DoN is particularly important in the manufacture of micro-structured NC membranes employed in lateral flow tests.^80^ By combining solvent/anti-solvent mixtures an NC porous membrane can be formed upon solvent evaporation, with pores located at the positions of the anti-solvent. This method gives important control over the membrane pore size, which in turn controls the flow rate of analyte across the membrane.^9^

The key variables that describe solubility through a ‘like-with-like’ solvent model can be summarised by the Hansen solubility parameters (HSPs);^58,81^ these are the dispersion forces, the dipolar intermolecular forces, and the hydrogen bonding interactions, all of which will vary with the DoN for NC. This approach has successfully been applied to identify suitable solvent mixtures for the 3D printing of NC with variable DoN,^82^ and to develop novel “green” solvents for NC lacquers.^83^ This is despite inconsistent reports of HSPs for NC,^82^ which are thought to arise because of difficulties in precisely and consistently determining the point of NC dissolution.

### 3.2 NC as a colloid suspension

While NC does not form micelles or well-defined particles in solution, the colloidal model of NC has nonetheless helped to rationalise the solution properties of NC. Urbanski suggested that NC can be considered a lyophilic colloid in solution,^6^ a property often associated with molecules comprising long polymer chains and large non-ionic polar groups, such as nitrate esters, that exhibit stronger solute–solvent interactions than solute–solute interactions.^84^ The three defining features of a lyophilic colloidal system, and the supporting evidence listed for its application to NC, are:

• Swelling with solvent prior to dissolution. NC will swell on exposure to solvents, as the solvent molecules interact strongly with the polymer chains to become incorporated into the material.^79^

• Forming viscous solutions even at low concentration. As a bulky polymer, NC will resist movement in solution, and strong interactions with solvents will decrease the diffusion rates of both solutes and solvents, making the solution viscous even at low concentrations.^8^

• No precipitation from saturated solutions of just one solvent. Concentrated solutions of NC become more viscous until they become gelatinised without precipitating. This again can be attributed to the strong solute–solvent interactions.

The swelling of NC *via* interpenetration of small molecules has been widely explored, much like the solution properties of NC. Many early studies worked to compare the swelling “power” of certain solvents and solvent mixtures, reporting that NC samples swelled over 13 times their weight in the presence of some solvents.^79^ Acetate and phthalate motifs were identified as swelling NC significantly whilst visibly preserving the macrostructure of the NC. Although highlighting the variable, but generally strong, interactions of NC with polar aprotic solvents, the methodology of this work is flawed as arbitrary boundaries need to be drawn to delineate between a highly swollen or gelled solid material. From this work it is clear that NC can take on high proportions of solvent whilst remaining as a solid, but the mechanism for penetration and the location of solvent molecules within the NC structure has not been proven.

### 3.3 Structure in swelled NC

Solvent-swelled NC forms thermodynamically stable pastes, with the double base propellant (DBP) formulation of NC combined with nitroglycerin (NG – a highly shock-sensitive liquid oxidiser) being a good example of this.^4^ There is evidence of molecular scale combination of NC and NG in DBPs by simply considering the changes in properties of each component:^85^ NC becomes plasticised, as observed by a decrease in its glass-transition temperature,^86^ and NG becomes de-sensitised, and significantly safer to handle. In an effort to explore this material at the molecular level, Ma *et al.* have reported MD simulations which indicated that non-classical (NC)C–H⋯ONO_2_(NG) hydrogen bonding interactions and significant van der Waals interactions exist between the two molecules.^87^ While this study can help explain the distinct properties of NC/NG mixtures, the NC model used was initiated from Happey's 5-fold helix model of NC that was based on flawed assumptions.^70^ As the NC chain conformation is critical to intermolecular interactions, the starting structure of the polymer could influence the results of these simulations. Further MD studies on NC^88,89^ have similar foundations, and while this illustrates the potential of MD simulations to predict mechanical properties, such as diffusion coefficients and material compressibility, there is scope for the improvement of such studies with better founded input structures.

Despite the high level of combination between NC and NG observed in DBPs, the fibrous structure of NC is preserved. This imparts the strong mechanical properties required for a material that is subjected to high-temperature and high-pressure operating conditions.^4^ NC fibres are optically anisotropic, as the refractive indices parallel and perpendicular to the fibre axis differ significantly.^47^ This results in negative birefringence, which can be observed by polarised light microscopy. Upon swelling with NG, the negative birefringence effect for NC becomes more pronounced, indicating anisotropy in the composite material has increased.^90^ This has been taken as evidence for both the preservation of the fibrous structure, and that the orientation of the polarisable nitrate groups of NG must lie perpendicular to the NC chain.

**Fig. 12:**
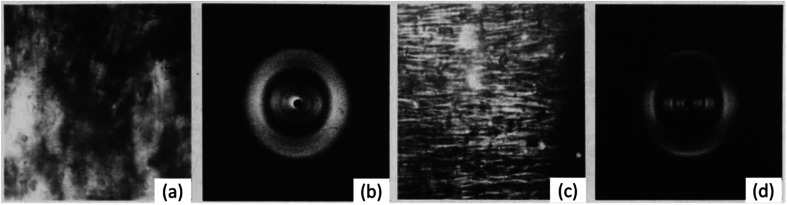
(a) Polarised light microscope images and (b) X-ray diffraction pattern of extruded fibres from mixed isotropic and anisotropic phases of NC/tetrahydrofuran/ethanol mixture. (c) Polarised light microscope images and (d) X-ray diffraction pattern of extruded fibres from completely anisotropic phase of NC/tetrahydrofuran/ethanol mixture. Reproduced from ref. ^92^ with permission from Taylor and Francis, copyright 1986.

So far, we have considered the structure of NC in its solid fibrous form. However, through the study of its liquid crystal (LC) form, the structure of NC in a solvated environment, similar to that of a swelled system, can be better understood. The general tendency for cellulose derivatives to form LCs has been attributed to the solvent-interacting side chains of the glucose monomer inhibiting rotation across the glycoside bridges.^91^ This therefore offers a route to induce artificial order into NC. Viney and Windle extruded fibres of NC from tetrahydrofuran/ethanol mixtures that were partially or fully anisotropic,^92^ and the resulting long-range order and anisotropy was studied through a combination of polarised light microscopy and X-ray fibre diffraction (see Fig. 12). If the extruded NC fibres were from partially anisotropic NC LCs, diffraction rings were observed, whereas the extruded fully anisotropic NC showed better molecular alignment and resulted in more obvious Bragg diffraction peaks. The latter indicated a high order in the lateral, inter-chain direction, despite the NC being partially nitrated. This was unexpected, as partially nitrated NC is believed to be a random sequence of variably nitrated monomers. Random co-polymers, such as co-polyesters, will in fact arrange based on their sequence of monomers inducing greater lateral order.^93^ Such matched sequences of monomers in the chain may be the mechanism by which order is induced in NC liquid crystals and their respective extruded fibres. The molecular interactions between chains have not yet been established, but this work shows the potential to produce reconstituted NC with a distinct molecular structure compared with that from the cellulose source. This could allow the structural influence of the initial cellulose source to be eliminated whilst retaining a synthetic fibrous structure. Future work, therefore, is required to characterise and compare the mechanical properties of synthetic NC fibres and the nitrated native cellulose fibres.

### 3.4 Surface interactions on NC

As a lacquer and coating, polymer surface interactions are a significant material property of NC. At medium degrees of nitration NC is wettable yet does not swell or become permeated by water, making it useful as a wood coating.^3^ Surface interactions on NC are perhaps most important in NC membranes as they are the most common immobilisation surface for biological materials^80^ and are used in modern high-throughput genomics methods.^94^ These porous membranes are optimised for each application based on their pore size as this influences the flow rate and absorption of analyte. Despite this, there are literature reports of NC membranes of the same pore size exhibiting different protein absorption and flow rate behaviours.^95^ As the microstructure and pore size are held relatively constant, it is likely that it is the variation in the material properties of NC that is responsible for this observation.

The current understanding of biomolecule absorption comes from just a few observations. Most assays are run below the isoelectric point of the protein, where the protein would be positively charged while the nitrate ester groups on NC would be negatively charged.^96^ Non-ionic surfactants can remove absorbed proteins from the surface of NC membranes, indicating that ionic interactions are not the only significant interaction between the analyte and the NC membrane. The other interaction is most likely hydrophobic in nature. By removing lipid moieties from glycolipids, Handman and Jarvis found that the lipid-free version did not adsorb onto NC membranes.^97^ Farrah *et al.* proposed that some combination between these two interactions occurs, such that at high pH hydrophobic interactions are dominant and at low pH ionic interactions dominate.^98^ While there is some understanding of the biomolecular surface interactions on NC, these have not been combined with understanding the material properties of NC. Even well-understood properties such as DoN have not been experimentally tested to understand the influence on biomolecular absorption. The surface properties of NC are also poorly understood, namely which functional groups in NC are most available at the polymer surface. For cellulose, there are distinct hydrophobic and hydrophilic edges which dictate its surface properties. Understanding the surface properties of NC would clearly be applicable to the design and choice of NC membranes for biomedical application and also for the widely used NC-based surface coatings.

## 4 Conclusions

This review has considered the structure of NC from production through to crystal structure as well as in composite materials where NC finds most of its applications. The choice of cellulose source material and nitration route have significant influences on the resulting structure and properties of NC. Good understanding of the mechanism of nitration helps control the DoN, a key material property of NC. Secondary agents, most commonly sulfuric acid, are key to the nitration process, but their high reactivity can also lead to hydrolysis and other side reactions of NC. The influence of these side reactions on the chain length and structure of NC remains unclear. This presents a barrier to the production of NC with defined structure and properties. The cellulose source also has a strong influence on the structure of NC through its nanostructure or proportions of natural impurities such as hemicellulose or lignin. Current commercial production of NC relies on plant sources known to vary in structure and purity with growth conditions. This variation is multi-faceted and, perhaps because of this, very little work has been conducted on determining the impact of such variation on the NC product.

Perhaps the most significant gap in our understanding of NC is the lack of a credible solid-state atomistic-level structure, which stem from the difficulties in obtaining high quality single crystals for diffraction studies for a material that is synthesised from a natural product polymer. Furthermore, NC is often used as a composite material, adding further levels of complexity when clarity at the atomistic level is sought. While early attempts were made, no new single crystal diffraction reports have been forthcoming since the 1970s, this despite the development of high flux X-ray radiation sources available at modern synchrotron facilities. Moreover, this early work was also carried out before the crystal structure of cellulose was fully determined; misunderstandings long since corrected in the cellulose literature have yet to be addressed for NC.

Changing the cellulose source, from plant to bacterial, offers a route to simplify research on NC, by replacing the uncontrollable environmental factors of the plant growth season with more regulated conditions achievable in a bioreactor. Reducing the variation within cellulose sources could allow NC to be manufactured more like a synthetic polymer, giving a standard by which samples could be more reliably compared.

Despite the inherent complexity, many relatively distinct areas of research have come together to give an understanding of NC which may be built into a more substantial and complete model. Greater understanding of cellulose biosynthesis, combined with limited but significant X-ray diffraction results, gives a model of NC which contains complex order and structure of the polymer chains. Considering NC in the context of colloids, polymer solubility and liquid crystals have not only rationalised experimental observations but has also given insights in its solvated structure. Well-founded models could give better control over the structure of NC, whether that be through adoption of bacterial cellulose as a precursor or through “synthetic” NC fibres ordered from anisotropic solutions. Such alternative NC materials offer good promise of a homogeneous, well-understood base material from which structure–property relationships could be drawn, allowing optimisation of such materials. This would not only make the study of NC easier, but it could also directly benefit its applications where homogeneity and reliability are primary concerns.

## Conflicts of interest

There are no conflicts to declare.

## Supplementary Material
